# Beyond scaling: how brains reorganize to support higher intelligence

**DOI:** 10.3389/fnsys.2026.1847194

**Published:** 2026-07-01

**Authors:** Paul J. Werbos, David S. Wack

**Affiliations:** 1 Retired, Alexandria, VA, United States; 2 Policy and Planning Committees of Millennium Project, Institute of Electrical and Electronics Engineers-USA, Washington, DC, United States; 3 Center for Large-Scale Complex Systems & Integrated Optimization Networks, The University of Memphis, Memphis, TN, United States; 4 Department of Radiology, Jacobs School of Medicine and Biomedical Sciences, University at Buffalo, Buffalo, NY, United States; 5 Department of Biomedical Engineering, University at Buffalo, Buffalo, NY, United States

**Keywords:** artificial intelligence, comparative neuroanatomy, cortical energy model, corticothalamic timing, white matter

## Abstract

Systems neuroscience—from Lashley's distributed engrams through Pribram's field-based processing to Freeman's oscillatory dynamics—has long argued that intelligence is a whole-brain property requiring feedback-driven computation. We formalize this tradition using Reinforcement Learning and Approximate Dynamic Programming (RLADP) and propose that vertebrate intelligence falls into four qualitatively distinct levels—rodent, primate, human, cetacean—each defined by a different architecture for generating and propagating backpropagated feedback signals. The transition between levels is not parametric but architectural, and each architecture demands a different energy strategy. A conserved allometric rule for cortical ion channels holds across nine of 10 mammalian species, fixing the biophysical cost of computation per unit volume; human neurons uniquely violate this rule, reducing channel density to redirect energy toward long-range white matter connectivity. We show that white matter is an active communication system whose superlinear scaling creates a geometric cost trap, that the corticothalamic loop provides master timing for forward-backward cortical processing cycles, and that timing degradation causes qualitative intelligence failure. The biological strategies cataloged here—from selective connectivity reduction to cellular energy reallocation to cortical reorganization—have parallels with the communication-energy wall now constraining artificial intelligence.

## 1 The intelligence hierarchy

Systems neuroscience—the study of how brains work as whole systems rather than as collections of specialized parts—has a deeper history and more radical claims than most contemporary neuroscience acknowledges. Lashley (1929, 1950) spent decades searching for the engram, the specific cortical location where a learned memory ought to be stored, and conclusively failed to find it . No matter where he lesioned rat cortex, learned behaviors degraded in proportion to how much tissue was removed, not which tissue was removed. The failure to localize the memory was itself the discovery: learned representations are distributed across cortex.

K.H. Pribram, Lashley's student, pursued the mechanism behind this distributed competence. His holonomic brain theory proposed that cortical processing operates through distributed interference patterns analogous to holograms—field-based representations rather than point-to-point wiring. In *Brain and Perception*, Pribram (1991) developed a comprehensive alternative to models that reduced neural computation to axonal outputs and synaptic weight changes, emphasizing instead the role of dendritic microprocesses and the electromagnetic field environment in which neurons operate. Freeman's (1995) extended this tradition in *Societies of Brains*, arguing that intentional action and social coordination, mediated by large-scale oscillatory dynamics, are the neural foundations of communication and meaning. Together, Lashley, Pribram, and Freeman represent the main line of systems neuroscience: intelligence is a whole-brain property, not a modular one, and understanding it requires mathematical frameworks adequate to distributed, feedback-driven computation (Werbos and Davis, 2016).

The behaviorist program of Skinner (1938) represented the opposite commitment: universal learning laws differing across species only in parameters, justifying decades of rat research as proxy for human behavior. M.E. Bitterman broke this assumption. His comparative experiments across vertebrate classes—fish, turtle, pigeon, rat, monkey—demonstrated qualitatively different *kinds* of learning. When given the same reversal-learning task, rats showed progressive improvement across reversals while fish did not, regardless of training. The differences between species are not parametric (Bitterman, 1965); they are architectural—cortical.

The intellectual roots of the required mathematical framework predate both behaviorism and systems neuroscience. Freud (1895)'s *Project for a Scientific Psychology* described cathexis—neural energy governed by pleasure and unpleasure—flowing through contact barriers of variable resistance, with learning requiring that both the presynaptic and postsynaptic neuron be influenced during excitation. Pribram, in his endorsement of *The Roots of Backpropagation* (Werbos, 1994), recognized this as an anticipation of backpropagation—the formal algorithm Werbos had developed in 1974. The formalization of backpropagation for neural networks (Werbos, 1974) was later generalized into Reinforcement Learning and Approximate Dynamic Programming (RLADP)—a framework in which backpropagation is a special case, and intelligence is optimization under uncertainty over time. RLADP provides the mathematical language that Lashley lacked and Pribram sought: a formal account of how distributed, feedback-driven systems can learn, optimize, and maintain coherence across the whole brain.

**Architecture, not intelligence**. This paper describes architectures, not cognition. Architecture is operationalizable across species — laminar structure, ion channel densities, white matter geometry — while cognition is the cultural overlay that runs on top (related to Dehaene and Cohen, 2007's cortical-recycling dynamic, though the underlying functions largely persist — humans retain face recognition after learning to read). The substrate is far older than the overlays we usually credit it for: anatomically modern *Homo sapiens* has existed for roughly 200,000 years (McDougall et al., 2005). In comparison, writing was invented by the Sumerians around 3,200 BCE (Schmandt-Besserat, 1992; Woods, 2010). And where we have direct evidence, the substrate has held still while the overlay has moved — IQ rose by approximately 30 points across the twentieth century on a substrate that did not biologically change (Flynn, 1987). Developing an overlay physically rewires the brain: six months of literacy training in previously illiterate adults remodels white matter and reorganizes subcortical structures previously thought to be developmentally fixed (Carreiras et al., 2009; Skeide et al., 2017). Some cognitive operations exist only once an overlay has developed: Ildefonso, a deaf man who acquired his first language in his late 20s, retained planning and emotion throughout and newly gained the capacity for abstract relational reasoning with language (Schaller, 1991). None of this happens without time — overlay depth accumulates across generations, so longevity is the biological condition that enables it, consistent with the grandmother hypothesis (Hawkes et al., 1998; Hawkes, 2003), which has independent empirical extension to cetacean matriarchs (Brent et al., 2015). And the apparatus we use to assess all of this is itself an overlay: the conscious narrator that reports on cognition is part of the verbal overlay (Hadamard, 1945). Human cognitive supremacy was first declared around the same time humans developed the overlays (writing and arithmetic) that produce the tests on which humans look supreme (White, 1967), and on a test outside those overlays — sequential numerical memory — Ayumu the chimpanzee beat naive adult humans (Inoue and Matsuzawa, 2007). The same overlay pattern is visible from the engineering side, where across a wide range of tasks large language model capability is dominated by the training corpus running on the architecture, not by the architecture alone (Banko and Brill, 2001).

We therefore rank architectures and decline to rank cognition. Each level described below represents a different architecture for generating and propagating feedback signals — the backpropagated value information that holds a learning system together — and each requires a different energy strategy to sustain its feedback loops. The transitions between levels are not quantitative; they are architectural — not a ladder of intelligence but a sequence of distinct strategies for sustaining feedback loops at increasing scale.

## 2 Four levels, four energy strategies

### 2.1 The conserved building plan

**Figure 1:**
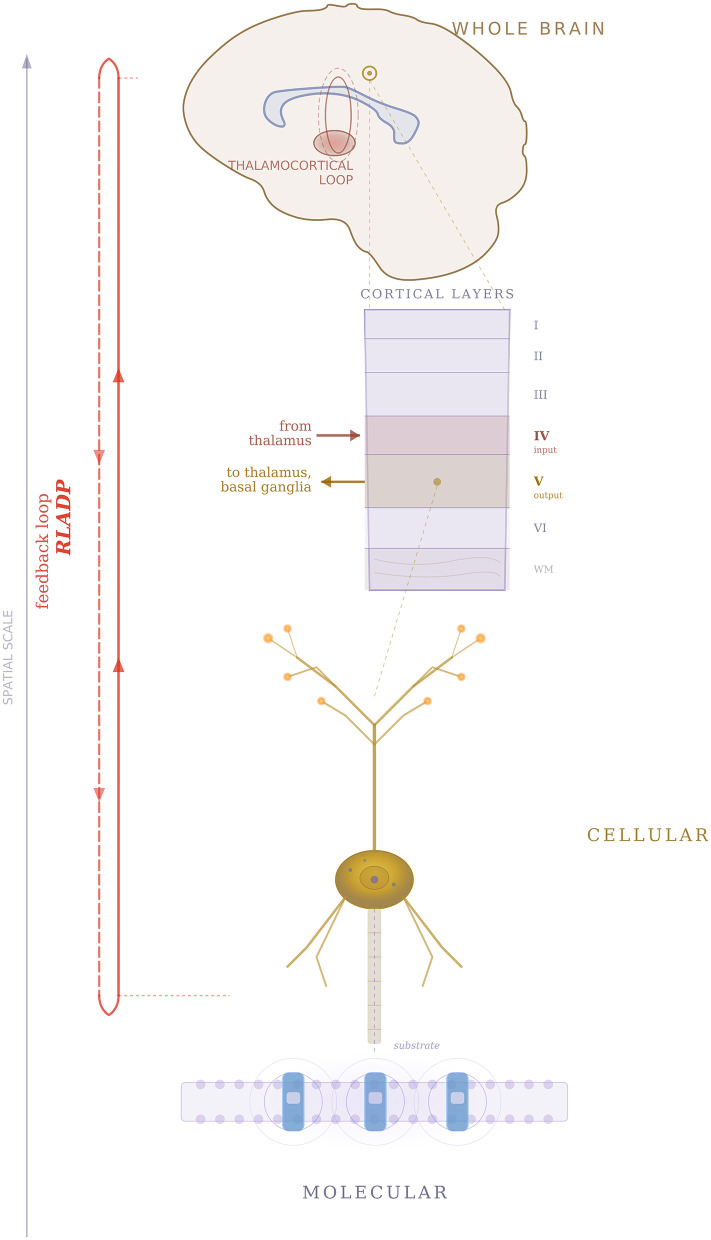
Coherence across scales. Progressive cutaway from whole brain to molecular substrate, illustrating the spatial hierarchy of the RLADP feedback architecture. **Top:** The corticothalamic loop provides master timing for forward-backward processing cycles. **Middle:** Cortical layers show thalamocortical input arriving at Layer IV and output departing from Layer V pyramidal neurons to thalamus and basal ganglia. A single Layer V pyramidal cell is expanded to show the dendritic arbor through which backpropagated error signals and apical timing inputs converge. **Bottom:** The molecular substrate—conserved ion channel arrays whose density follows a universal allometric rule in all mammals except humans (Beaulieu-Laroche et al., 2021). The red dashed loop (left) marks the spatial extent of the RLADP feedback circuit, spanning whole-brain and cellular levels; the molecular level provides the biophysical substrate enabling computation but is not itself part of the feedback loop.

One foundational fact must be established before describing the hierarchy. Beaulieu-Laroche et al. (2021) measured ion channel properties in layer five cortical pyramidal neurons across ten mammalian species spanning a 14,000-fold range in brain size, from Etruscan shrew to human. They discovered a conserved allometric rule: species with larger neurons compensate by packing more ion channels into each unit of membrane surface, scaling in precise proportion to the decreased surface-to-volume ratio of larger cells. The result is that the total ionic current capacity per unit volume of cortex is constant across species, meaning the biophysical cost of computation per cubic millimeter of brain is the same regardless of brain size. Nine of the 10 species studied follow this rule. Humans are the exception, and that exception is the key to Level 3. Figure 1 illustrates this architecture as a progressive cutaway from corticothalamic loop to cortical layers, pyramidal cell, and molecular substrate, showing how the RLADP feedback circuit spans whole-brain and cellular levels while resting on the conserved ion channel plan. The feedback circuit operates on neural signaling variables — spike timing, synaptic activity, value estimates — at neural-signaling timescales; the ion channel substrate enables these dynamics but is not itself a set of variables over which the algorithm operates.

### 2.2 Level 1—Rodent

Level 1 represents the base mammalian architecture: reinforcement learning via approximate dynamic programming, with basal ganglia implementing hierarchical decision structures—verbs (discrete action choices), objects (multiplexed representations), and modifiers (continuous parameters)—within a standard six-layer neocortex (Werbos, 2009). Learning is first-person only—the organism can learn from its own experience but not from observing others. Neurons follow the conserved building plan described above. With small brains and short white matter distances, the geometric communication cost described in Section 3 does not yet impose a hard constraint. This is the architecture most current AI replicates.

### 2.3 Level 2—Primate

Level 2 introduces a fundamentally new architecture, not a parameter upgrade. Mirror neurons, systematically characterized and named in macaque premotor cortex by Gallese et al. (1996), fire both when the animal performs an action and when it watches another individual perform the same action, creating a neural bridge between self and other. The organism can now learn from watching, not just from doing. Macaque neurons still follow the conserved ion channel density rule (Beaulieu-Laroche et al., 2021), so the transition from Level 1 to Level 2 is a wiring change, not a cellular one. The additional communication cost of connecting a larger brain is partially managed because primate white matter scales more slowly with neuron number than rodent white matter, with an exponent of approximately 1.08 vs. 2.01 (Ventura-Antunes et al., 2013), a difference driven by cortical folding reducing required connection distances (Mota et al., 2019). In effect, primates partially escape the geometric cost trap by reducing the fraction of neurons that maintain long-range connections as brain size increases—a strategic retreat from full connectivity.

### 2.4 Level 3—Human

Human layer 5 pyramidal neurons are the sole exception to the conserved building plan. They have far lower densities of voltage-gated potassium channels and HCN (hyperpolarization-activated cyclic nucleotide-gated) channels—the channels that regulate resting membrane potential and dendritic signal integration—than the allometric scaling from other species predicts (Beaulieu-Laroche et al., 2021). In every other species, larger neurons compensate for their lower surface-to-volume ratio by increasing ion channel density proportionally. Human neurons do not make this compensation, which means each human neuron requires less energy to maintain its resting state and to fire than a species-general model would predict. Additionally, human dendrites—the branching input structures of neurons—are more electrically compartmentalized than in other species: individual dendritic branches can generate and sustain their own local voltage responses independently of the cell body, rather than simply funneling all input to a single integration point (Beaulieu-Laroche et al., 2018). In practical terms, a single human neuron can perform some of the computational work that would require multiple neurons in other species. The energy saved by reduced ion channel costs may be reinvested in long-range white matter connectivity—in the human brain, whole-brain white matter volume is roughly 77% of total grey matter volume by midlife (Irimia, 2021), a ratio far higher than in smaller-brained species. The transition to Level 3 required not just new wiring but a new cellular energy strategy: spend less on each neuron, spend more on communication between neurons.

This energy reallocation underwrites a qualitative expansion of the mirror system. Humans extend mirror neuron capabilities to act out past experience—through gesture, dance, and eventually language—so others can observe and encode it via their own mirror systems. Werbos (2012) argued that language arose not as innate Chomskyan grammar but as a learned dance of words built on the ability to perform experience for a mirroring audience—a view compatible with Freeman's (1995) account of communication as rooted in intentional action and social coordination.

### 2.5 Level 4—Cetacean

Cetacean cortex is not a scaled-up version of the terrestrial mammalian plan; it is a structurally distinct organ. Layer IV—the primary thalamocortical input layer in all terrestrial mammals—is completely absent, and large inverted neurons in the cell-dense layer II replace the standard input architecture, requiring a fundamentally reorganized routing of sensory information (Hof and Van der Gucht, 2007; Marino et al., 2007). Pilot whale neocortex contains approximately 37 billion neurons (Mortensen et al., 2014), roughly twice the 16 billion cerebral cortical neurons in humans (Azevedo et al., 2009), housed in a cortex that is thinner but far more extensively folded than the human version. Von Economo neurons—large, spindle-shaped cells concentrated in humans in anterior cingulate and frontoinsular cortex—appear in cetaceans in these same regions plus frontopolar cortex, in numbers comparable to those of great apes (Butti et al., 2009). The gene turnover rate across the cetacean genome runs more than three times faster than in non-cetacean mammals, with a disproportionate share of ion channel genes under positive selection (Uribe et al., 2024). The architecture also extends beyond the individual: cetacean pods operate as distributed acoustic sensing arrays, with non-emitting animals extracting object information from clicks emitted by other pod members (Xitco and Roitblat, 1996), and every emission carries individual identity through learned signature whistles whose frequency contour conveys identity independent of voice features (Janik et al., 2006). The functional architecture at Level 4 remains the least empirically constrained in this hierarchy—no functional imaging or electrophysiology has been conducted on a cetacean brain during complex behavior.

No lineage reached a higher level by adding more of the same. Level 1 to 2 is a wiring change. Level 2 to 3 requires cellular reorganization to shift the energy budget from computation to communication. Level 3 to 4, as far as the anatomical evidence indicates, requires rebuilding the cortical architecture itself—new laminar organization and positive genetic selection on the ion channel genes that define what neurons can do.

## 3 The cost of connection

Zhang and Sejnowski (2000) established a scaling law: across 59 mammalian species, white matter volume scales as grey matter volume raised to the power 1.23. Every increase in computation (grey matter) demands a superlinear increase in communication infrastructure (white matter). The reason is geometric: grey matter is a two-dimensional computational sheet approximately 2–4 mm thick, while white matter fills the three-dimensional volume beneath it. Connecting points on a surface through a volume imposes cubic scaling costs. In whale and elephant brains, white matter volume approaches 80% of grey matter volume; in the smallest insectivores the ratio drops below 7% (Zhang and Sejnowski, 2000). As brains scale up, communication infrastructure balloons from a negligible fraction of cortical volume toward near-parity with the computational sheet itself.

Standard fMRI practice has been to restrict statistical analysis to grey matter voxels, masking out white matter on the assumption that it passively conducts signals while only grey matter computes. That assumption is now untenable. Task-evoked BOLD responses in white matter have been demonstrated across multiple tract systems (Gawryluk et al., 2014; Schilling et al., 2023). Intracranial stereoelectroencephalography (SEEG) confirms that white matter BOLD signal correlates with direct electrophysiological measures of neural synchronization, providing ground truth that the signal is real (Huang et al., 2023). As (Grajauskas et al. 2019) argued, white matter fMRI activation should not be masked out or ignored. Our earlier work (Wack et al., 2012, 2014) demonstrated this directly: in a binaural listening task requiring precise interhemispheric timing, fMRI revealed activation within the corpus callosum itself, and DTI measures of white matter tract microstructure within the brainstem correlated strongly with signal detection thresholds, supporting that white matter structural integrity predicts functional performance in timing-dependent tasks.

Communication between neurons also extends beyond synaptic transmission. When neurons are active, the flow of ions across their membranes generates extracellular electrical fields—the source of the EEG signal—that can feed onto nearby neurons through ephaptic coupling, direct field-to-membrane influence without any synaptic connection (Buzsáki et al., 2012). Anastassiou et al. (2011) demonstrated that even fields producing membrane potential changes of less than 0.5 millivolts can entrain the timing of action potentials in nearby neurons. Whether ephaptic coupling at this scale aggregates into population-level computation remains debated (Anastassiou and Koch, 2015), but the cellular-level effect itself — coordination across cells by extracellular field rather than synaptic contact — is the kind of mechanism Pribram argued was missing from connectionist models: a field-based coordination layer operating alongside synaptic transmission.

Communication is not free. Every spike propagating down an axon requires ion channel activity at every node of Ranvier—the gaps in the myelin insulation where the signal is regenerated—consuming ATP and demanding blood flow. White matter energy cost compounds: superlinear volume scaling sets the structural price, and traffic intensity sets the metabolic one.

## 4 Timing is everything

Connection alone is not sufficient for intelligence; signals must also arrive at the right time.

Jeffress (1948) proposed a foundational model: dedicated neural delay lines and coincidence detectors that convert interaural time differences (ITDs) into a spatial map of sound location. The mammalian auditory system can resolve ITDs as small as 10 μs (Klumpp and Eady, 1956; Grothe et al., 2010)—50 to 100 times finer than the approximately 1 ms duration of a typical action potential (Bean, 2007). The system achieves precision far below the resolution of its individual components, but depends critically on the integrity of every link: synapses, axons, myelin, and the interhemispheric connections that coordinate inputs from the two ears.

Interhemispheric communication relies on the corpus callosum, which is not a uniform cable but a delay distribution system: conduction delays vary with both axon diameter and connection length, spanning from a few milliseconds for thick motor fibers to more than 15 ms for thin prefrontal fibers in the human brain (Caminiti et al., 2013). Callosal fiber composition varies by species and region, tracking timing requirements: sensory regions demanding microsecond precision are ~94% myelinated with fast-conducting axons, while association regions are only ~70% myelinated (Olivares et al., 2001; LaMantia and Rakic, 1990).

This principle extends beyond auditory processing to cortex itself. Werbos and Davis (2016) used spike-sorted and burst-sorted data from the Buzsáki laboratory (Fujisawa et al., 2008) to test a prediction of the RLADP framework: that cortical pyramidal cells alternate between a forward computational pass and a backward error-propagation pass within each clock cycle, with the nonspecific thalamus providing the master timing signal via inputs to apical dendrites (Werbos, 2009) (Figure 1). The empirical analysis found that the firing sequence in the second half of each ~153 ms cycle more closely resembled a mirror image of the first half than a repetition of it—consistent with forward-then-backward processing in this dataset. The error in assuming mirror-image propagation was approximately one-third less than the error in assuming repetition. The theory further proposes coupled cortical computation and limbic evaluation clocks, with the limbic system assessing cortical output quality through the basal ganglia. If supported by replication across cortical areas and species, this organization would provide temporal structure for the RLADP architecture in cortex: one clock for computation, one for evaluation.

These cycles depend on the same infrastructure as auditory timing—myelin integrity, callosal transmission, and cortico-thalamo-cortical synchronization loops that produce zero-lag coordination between hemispheres (Vicente et al., 2008). If these cycles represent the temporal substrate of backpropagation-like processing in the living brain, then timing precision is what the brain's energy budget is ultimately buying. Before failure, the system may compensate—rerouting through costlier pathways at chronic metabolic expense (Wack et al., 2025), consistent with the broader principle that neural systems recruit additional resources to maintain performance against substrate degradation (Reuter-Lorenz and Cappell, 2008). When timing degrades—through demyelination, axonal loss, or disconnection—intelligence does not merely slow down; it fails qualitatively because the feedback signals that hold the system together no longer arrive when they are needed.

## 5 Implications

### 5.1 The AI communication-energy wall

Current AI faces the same cost structure, though the mechanisms differ. In biology, the white matter geometric trap is a physical volume constraint: connecting points on a two-dimensional cortical sheet through three-dimensional space imposes superlinear scaling on communication infrastructure. In transformer architectures, *O*(*n*^2^) self-attention is a computational constraint: every token attending to every other imposes quadratic scaling on memory and processing. In both cases, the cost of communication grows faster than the cost of computation, and at sufficient scale, communication cost becomes the binding constraint on further expansion.

The biological strategies cataloged in this paper suggest artificial analogs. Distilled models stay small, avoiding the trap by limiting the number of elements that must communicate (rodent strategy). Sparse attention and mixture-of-experts architectures reduce the fraction of elements maintaining long-range connections as the system grows (primate strategy). Heterogeneous compute with efficiency cores reallocates energy from uniform processing toward targeted communication (human strategy). Non-transformer architectures such as state space models reject the all-to-all computational plan entirely, representing a fundamentally different substrate analogous to the bird strategy discussed below. No current AI analog exists for the cetacean approach of rebuilding the architecture itself while keeping communication distances short despite large element counts. The hierarchy proposed here provides a development roadmap: Level 1 to 2 requires modeling other agents. Level 2 to 3 requires energy reallocation at the substrate level. Level 3 to 4 requires redesigning the basic computational units and their connectivity plan.

### 5.2 Bird diversity

The mammalian hierarchy is not the only path to complex intelligence. Birds achieve complex cognition using pallium-based computation with no neocortex and no grey-white matter segregation as mammals know it. Corvids transmit threat identity to naive group members across generations, plan counted vocalizations, and show face-selective PET activation (Marzluff et al., 2010, 2012; Cornell et al., 2012; Liao et al., 2024). Whether bird intelligence maps onto the hierarchy proposed here or represents a fundamentally different set of architectural solutions remains an open question.

### 5.3 Beyond classical architectures

The hierarchy described above covers architectures operating within classical electrodynamics. Yet one conspicuous gap remains unexplained: the energy efficiency of biological neurons exceeds anything achieved in artificial hardware by orders of magnitude, and the conserved building plan of Beaulieu-Laroche et al. (2021) does not fully account for how neurons maintain such efficiency at the molecular level. The possibility that quantum effects within individual neurons contribute to this efficiency has motivated sustained investigation, from the quantum molecular computing model of Conrad (1992) to the quantum field approach of Jibu and Yasue in Pribram's *Brain and Perception* (Pribram, 1991). The RLADP framework can be extended to quantum substrates: just as classical backpropagation optimizes over classical state spaces, quantum extensions of approximate dynamic programming can optimize over superposition states, potentially opening computational capabilities inaccessible to classical machines (Werbos, 2024). Whether biological brains exploit such capabilities remains an empirical question. The mathematical framework — including the granted thermal quantum annealing methods (Werbos, 2024) and their integration with Quantum RLADP and quantum matrix methods — is independently developed and does not depend on the biological hierarchy described above.

## Data Availability

The original contributions presented in the study are included in the article/supplementary material, further inquiries can be directed to the corresponding author.
